# Variation in tissue-specific gene expression among natural populations

**DOI:** 10.1186/gb-2005-6-2-r13

**Published:** 2005-01-26

**Authors:** Andrew Whitehead, Douglas L Crawford

**Affiliations:** 1 Rosenstiel School of Marine and Atmospheric Science, University of Miami, 4600 Rickenbacker Causeway, Miami, FL 33149, USA

## Abstract

**Background:**

Variation in gene expression is extensive among tissues, individuals, strains, populations and species. The interactions among these sources of variation are relevant for physiological studies such as disease or toxic stress; for example, it is common for pathologies such as cancer, heart failure and metabolic disease to be associated with changes in tissue-specific gene expression or changes in metabolic gene expression. But how conserved these differences are among outbred individuals and among populations has not been well documented. To address this we examined the expression of a selected suite of 192 metabolic genes in brain, heart and liver in three populations of the teleost fish *Fundulus heteroclitus *using a highly replicated experimental design.

**Results:**

Half of the genes (48%) were differentially expressed among individuals within a population-tissue group and 76% were differentially expressed among tissues. Differences among tissues reflected well established tissue-specific metabolic requirements, suggesting that these measures of gene expression accurately reflect changes in proteins and their phenotypic effects. Remarkably, only a small subset (31%) of tissue-specific differences was consistent in all three populations.

**Conclusions:**

These data indicate that many tissue-specific differences in gene expression are unique to one population and thus are unlikely to contribute to fundamental differences between tissue types. We suggest that those subsets of treatment-specific gene expression patterns that are conserved between taxa are most likely to be functionally related to the physiological state in question.

## Abstract 2

The expression of a selected suite of 192 metabolic genes in brain, heart and liver in three populations of the teleost fish *Fundulus heteroclitus* was examined. Only a small subset (31%) of tissue-specific differences was consistent in all three populations, indicating that many tissue-specific differences in gene expression are unique to one population and thus are unlikely to contribute to fundamental differences between tissue types.

## Background

The regulation of gene expression varies extensively among tissues, individuals, strains, populations and species [1-6] and variation in gene expression has a genetic basis [7,8]. Despite such biological variance, differences in gene expression are used to describe cancers [9-12], heart failure [13,14] and metabolic diseases [15]. It is common for these pathologies to be associated with changes in tissue-specific gene expression or changes in metabolic gene expression. For example, many different cancers have unique tissue-specific patterns of gene expression [16], and thyroid cancers are associated with increases in aerobic metabolic gene expression [17].

Although tissue-specific gene expression patterns are often used as a method to identify functionally relevant genes, how conserved these differences are among outbred individuals and among populations has not been well documented. It is possible that many of these changes represent polymorphism among individuals or populations and are not specifically associated with disease. To address this we used a well established system (tissue-specific gene expression) and genes with well defined function and tissue-specific distributions (metabolic genes).

Given the high variance in gene expression among individuals and populations, our goal was to examine the conservation of tissue-specific gene expression among populations of the same species. Specifically, we assessed the among-population variance of tissue-specific patterns of gene expression (in brain, heart and liver) in the teleost fish *Fundulus heteroclitus*. A cDNA microarray was used to measure levels of expression in normal healthy male fish for 192 genes involved in central metabolic pathways. We used this compact array in order to impose a high degree of technical and biological replication (24 replicates for each of three tissues from nine individuals with two samples per array). Also, this array was used because metabolic genes are essential, are known to have tissue-specific expression, especially in fish, and are often misused as controls with little characterization of variation in expression among individuals or tissues. Analysis of variance (ANOVA) was used as a statistical test to determine which genes were differentially expressed among tissues and populations. Tissue-specific patterns of gene expression were compared among populations. As expected, we detected extensive variation in gene expression among tissues. Unexpectedly, only a fraction (31%) of tissue-specific differences was conserved between all populations.

## Results

### Variation among

**Figure 1:**
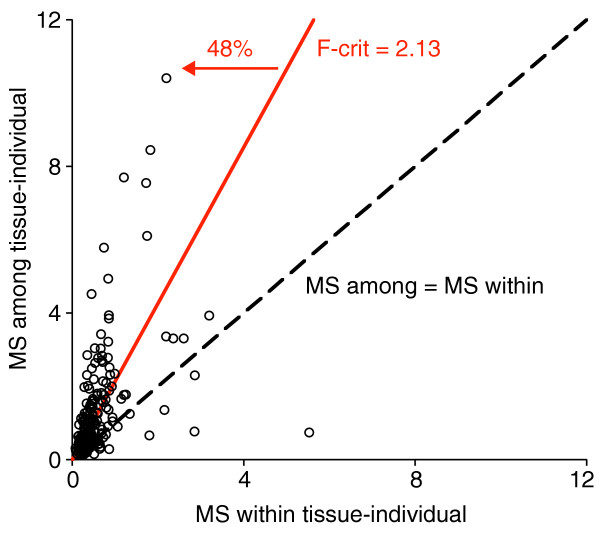
Variation within individuals (technical variance) and among individuals within populations and tissues (biological variance) for each of 192 genes indicated by the mean square error (MS) of measurements. Points above the dashed line indicate genes with greater variance among individuals than within. F-crit is the critical value of the F-statistic (F = MS_among_/MS_within_, with 12 and 27 degrees of freedom and α = 0.05) for testing significant differences in gene expression between individuals. For 48% of genes, MS_among_/MS_within _> F-crit (solid red line). These genes are therefore differentially expressed among individuals within treatments.

Variation among individuals within groups was high (groups included the nine tissue-by-population groupings; Figure 1). Nearly half of genes (92 genes, 48%) were differentially expressed (*p *< 0.05) among individuals within populations and tissues (Figure 1), and inter-individual differences ranged over fivefold.

### Variation among tissues

**Figure 2:**
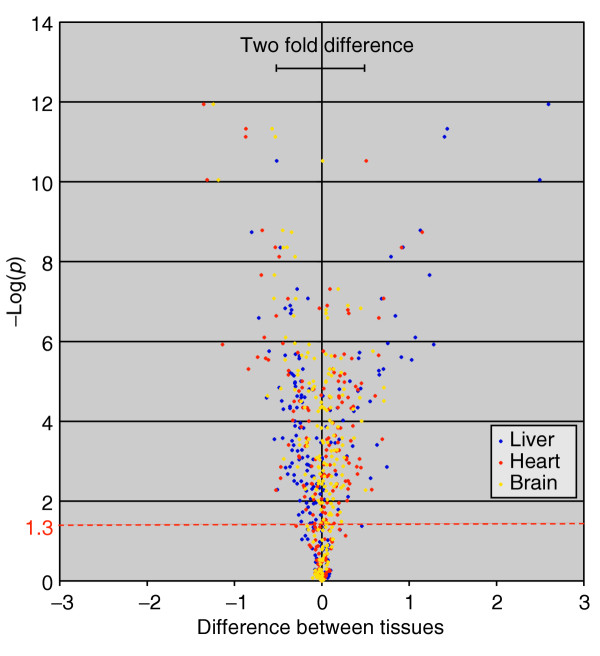
Volcano plot of differences between tissues and corresponding *p*-values. Differences in expression for each gene is the log_2 _ratio of tissue mean expression minus grand mean; a twofold difference in expression between tissues is indicated by one unit separation along the *x*-axis. *p*-values for differences in gene expression among tissues were calculated using ANOVA, and illustrated as -log(*p*). A *p*-value of 10^-4 ^is expressed as 4 on the *y*-axis, and the α = 0.05 threshold is indicated by the red dashed line (1 - log(0.05) = 1.3).

**Figure 3:**
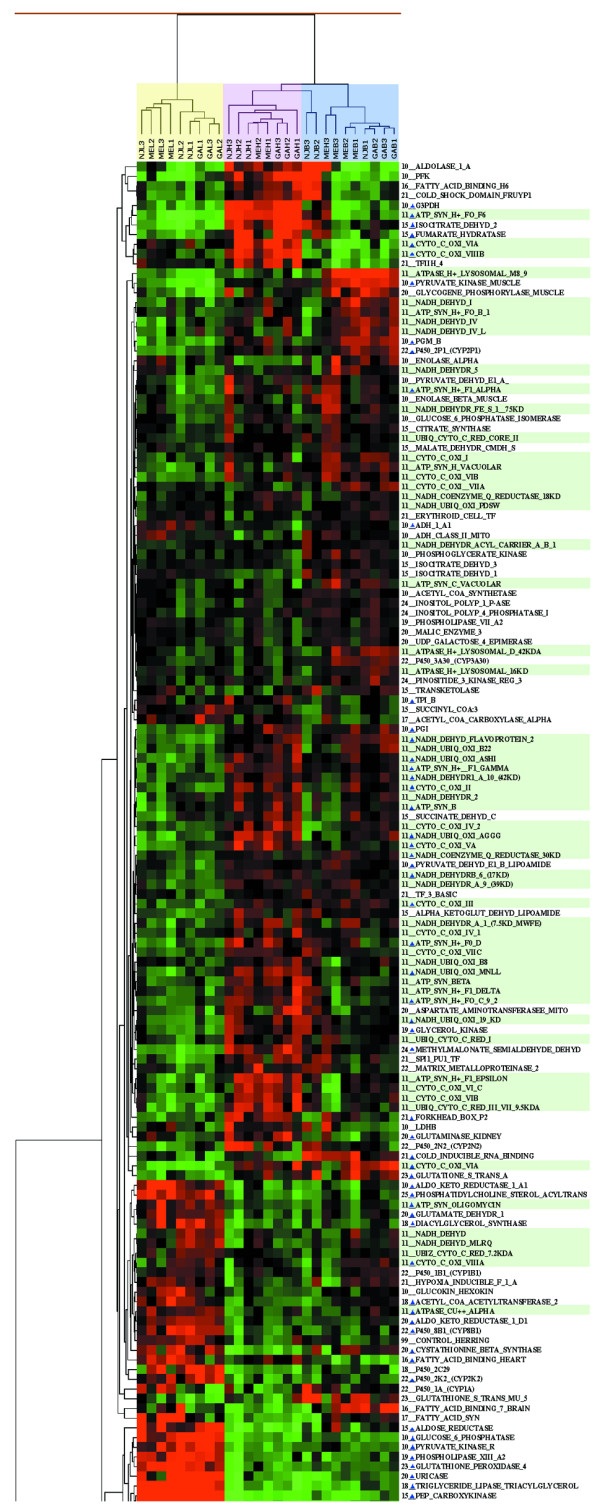
Dendrogram of gene expression patterns across samples for genes significantly different between tissues (ANOVA, *p *< 0.05). Clustering indicates similar expression patterns among samples (top axis) and among genes (left axis). Samples cluster as livers (yellow), hearts (pink) and brains (blue). Genes involved in oxidative phosphorylation are highlighted in green, and expression patterns that are consistent across all three populations are highlighted with a blue triangle.

Although variation among individuals was high, added variation due to tissues was significant. Considering 192 genes and a *p*-value of 5%, one would expect less than 10 false-positive differences among tissues under the null hypothesis. We detected 76% of genes (146 of 192 genes) differentially expressed among brains, hearts and livers (ANOVA, *p *< 0.05). Selecting the α level at which differences between treatments are considered significant is problematic because of the large number of comparisons performed. As such, we present a volcano plot to illustrate the range of expression differences between tissues and associated *p*-values (Figure 2). When α is set at 0.01, 0.001 or at the Bonferroni-corrected value (2.6 × 10^-4^), the proportion of significant genes is 67% (129 genes), 50% (96) and 39% (75), respectively. Significant differences in expression ranged from less than 1.2-fold to nearly 16-fold (Figure 2). The predominant pattern of tissue-specific expression can be described by expression significantly different in the liver compared to the other two tissues (Figure 3).

Many expected tissue-specific patterns emerged. For example, the brain-specific fatty-acid-binding protein was typically more highly expressed in the brain than in other tissues (*p *= 0.005), hepatocyte nuclear factor 4-alpha (a transcription factor) was more highly expressed in liver than in other tissues (*p *< 0.001), and two genes involved in glycerolipid metabolism -lipoprotein lipase and phopholipase XIII A2 - were more highly expressed in liver than other tissues (*p *< 0.001 for both genes).

**Figure 4:**
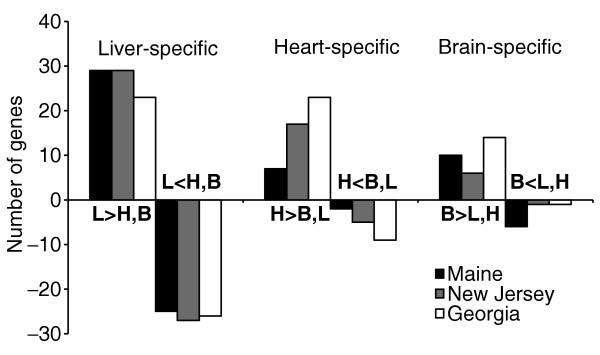
Number of genes differentially expressed among tissue groups for each population. Tissue-specific genes are those that are expressed more highly in a tissue than in the other tissues (for example, L > H, B) or lower in a tissue than in the other tissues (for example, L < H, B).

**Figure 5:**
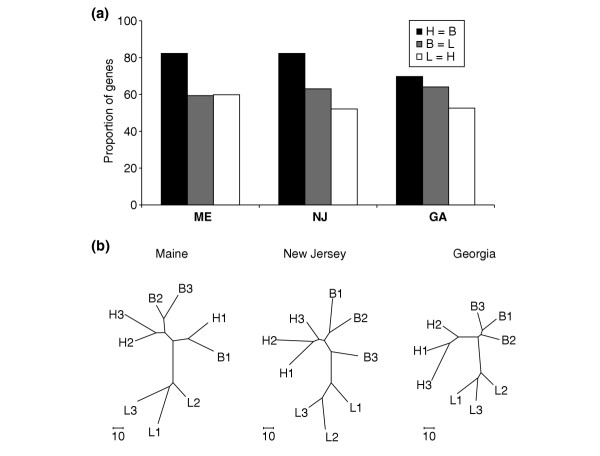
Similarity of expression patterns among tissues. **(a) **Proportion of 192 genes that are similarly expressed between heart and brain (black bar), brain and liver (gray bar) and liver and heart (white bar), for each population including Maine (ME), New Jersey (NJ) and Georgia (GA). **(b) **Neighbor-joining trees of global similarity of expression patterns among samples (L, liver; H, heart; B, brain) for each population. Distance between samples is the sum of differences of log_2 _expression values over all genes.

Liver-specific expression accounted for 61% of the expression differences among tissues (Figure 4). Heart-specific and brain-specific expression accounted for 24% and 15% of differences among tissues, respectively. Regardless of population, expression patterns were typically most similar between heart and brain, and least similar between liver and heart (Figure 5). There were 67 genes printed on the array that code for proteins involved in oxidative phosphorylation, and 88% (59 genes) were differentially expressed between tissues (genes highlighted in green, Figure 3). Of differentially expressed oxidative phosphorylation genes, only 10% (six genes) were expressed more highly in the liver than in other tissues, whereas the remaining 90% (53 genes) had lower expression in the liver compared to brain or heart.

### Variation among taxa

A small proportion of genes (six genes, 3%) differed in expression among populations (*p *< 0.05). However, it should be noted that although the split-plot design is powerful for detecting differences between split-plot factors (tissues), it is considered to have low power for detecting differences between blocks (populations) [18]. As such, it is likely that 3% is an underestimate of true among-population differences in gene expression. Indeed, two-way ANOVA (data not shown), which has higher power for detecting population differences but is less valid than the split-plot model for testing individual and tissue differences, detected among-population differences in expression for 18% of genes at *p *< 0.05, or 6.3% of genes at *p *< 0.01. Each tissue contributed a similar number of genes differentially expressed among populations.

**Figure 6:**
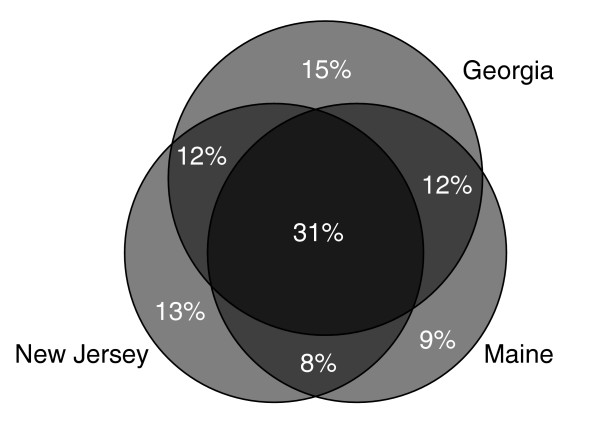
Shared expression patterns among populations.

Surprisingly, differences among tissues in gene expression were not consistent across all three populations. More than one-third (37%) of the genes differentially expressed between tissues were significant in only one of the three populations (Figure 6). Population-specific differences were distributed among the three populations; Georgia had 40% of the population-specific genes, and New Jersey and Maine had 34% and 26%, respectively. A proportion of these inconsistencies could be due to false-positive or false-negative differences between tissues in individual populations. However, statistically significant interaction between tissue and population was detected for many (30%) of these inconsistencies (see Additional data file 1).

**Figure 7:**
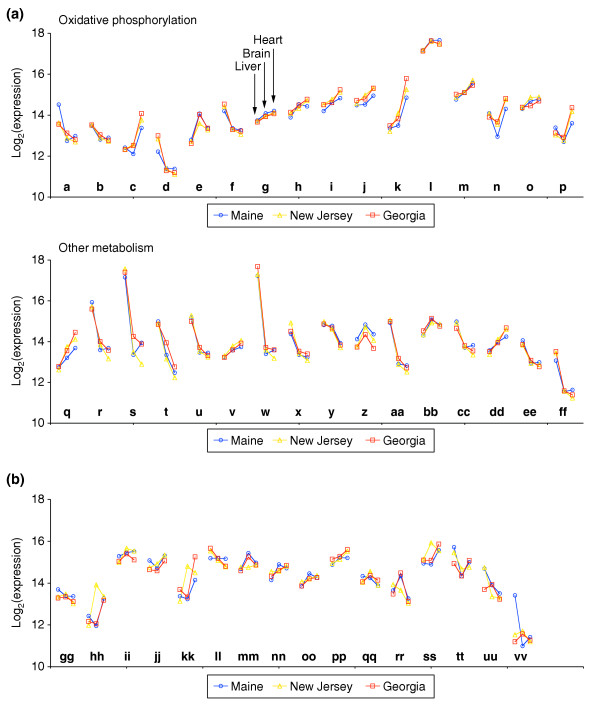
Gene expression in liver, brain and heart (three symbols for each line) for the three different populations (three lines per gene). Each letter represents a gene, expression values are log_2 _transformed and are indicated for liver, brain and heart (left to right) in each of Maine (circles), New Jersey (triangles) and Georgia (squares) populations. **(a) **Genes consistently different among tissues in all three populations are grouped as those involved in oxidative phosphorylation (upper panel) and those involved in other metabolic pathways (lower panel). **(b) **A representative subset of genes not consistently different among tissues in all populations. Gene names associated with letters are provided in Table 1 and Additional data file 1.

A relatively small proportion of tissue-specific genes (31%) have consistent expression patterns in all three populations (Figure 6; also see Additional data file 1 for details). This subset of genes also reflects the different metabolic status of brain, heart and liver; most of the genes involved in oxidative phosphorylation were more highly expressed in brain and heart than in liver (Figure 7a, Table 1), and most of the genes involved in fatty-acid metabolism, glycerolipid metabolism, steroid metabolism and detoxification were more highly expressed in liver. The majority of the tissue-specific genes were not consistent among populations (a subset of these genes are illustrated in Figure 7b, Table 1).

### Quality control

Variation among technical replicates was low, and permutation tests indicated that the ANOVA model was robust. Sample coefficients of variation (CVs (standard deviation/mean) × 100), which estimate technical variance due to replicate spots (six spots per hybridization), repeated measures (two hybridizations per dye), and dye (two dyes per sample), were calculated for each gene of each of the 27 samples. CVs less than 5% accounted for 95% of sample/genes, respectively. Of the many comparisons performed (differences among tissues, populations, interaction), permutation tests results agreed with ANOVA results (the same comparisons identified as significant or not significant) for 99.1% of comparisons, suggesting that our ANOVA model was robust.

## Discussion

Considerable variation occurs among the 27 samples (three tissues from each of three individuals from three populations) used to measure inter-individual and tissue-specific variation in gene expression. We are able to precisely describe the patterns of gene expression for 192 metabolic genes because of the low experimental variation; for 95% of the replicate measures of gene expression the standard deviation is less than 5% of the mean. Notably, gene expression is statistically different for many genes among individuals within a population for a tissue (48%), between tissues (76%), and between populations (3%). For genes with tissue-specific expression, only a fraction (31%) had expression patterns consistent across all three populations. These data do not specifically identify tissue-specific differences that are inconsistent across populations, but rather emphasize that tissue-specific differences detected can vary from one population to another. When measured from a single population, highly significant differences in tissue-specific expression do not necessarily represent genes relevant to general functional or morphological differences between tissues.

### Variation among individuals

Variation in gene expression among healthy male individuals raised under controlled laboratory conditions was high. Nearly half of the metabolic genes (48%) were differentially expressed among individuals within a population for any one tissue (Figure 1), with fold differences ranging from 1.2- to 5-fold and *p*-values ranging down to 10^-7^. Differences in gene expression among individuals are unlikely to be due to common reversible environmental factors that affect physiological performance (acclimation effects) since all individuals used in this study were housed in a common environment and fed the same food for at least two months. However, the differences could be due to irreversible developmental effects or genetic variations that affect gene expression. Regardless of this, if these differences are heritable or due to developmental plasticity, they represent variation one would expect to find among outbred organisms, including humans.

Other studies that have measured inter-individual differences in gene expression have also detected high levels of variation in a variety of taxa. Among crosses of different yeast strains a large number of differences in expression (6% of genes varying more than twofold) were detected between morphotypes [1]. A previous study of the same Maine and Georgia *Fundulus *populations assayed here detected 18% of genes differentially expressed among healthy individuals [3]. Although inter-individual variance in gene expression seems prevalent, our observation that 48% of genes are differentially expressed among individuals is high. This may reflect the greater precision of these measurements as a result of extensive technical replication (24 replicate measures per sample) as coefficients of variation for technical replicates was less than 5% for 95% of the genes. Indeed, using similar methods and tools, a concurrent study assessing variation in *Fundulus *also detected a very high proportion of genes (94%) differentially expressed among individuals [19]. Alternatively, since our array is heavily biased toward metabolic genes, detected variance may also reflect a greater variation in metabolic gene expression. We could speculate that the high variation in metabolic genes reflects a greater allowable variation. That is, there may be less selective pressure to constrain metabolic variation either because varying the amount of an enzyme does not affect metabolism or variation in metabolism is phenotypically acceptable. One could test this by using an array with more comprehensive representation of the genome and comparing variances of different gene classes defined by function.

Considering the high inter-individual variation detected, the data presented here underscore the importance of including biological replicates within treatment groups in order to ascribe differences in expression to treatment rather than to inter-individual variation. Statistically, an analysis of variance can be used to examine the effects of technical and biological variation, and these tests have proved powerful for detecting significant differences in gene expression [3,4], even differences as small as 1.2-fold. The cost of resources in microarray experiments should no longer excuse lack of biological and technical replication. Often, microarray experiments pool individual samples within treatment groups to capture biological variation. However, this approach only estimates an average level of expression and fails to estimate biological variation. When only small quantities of RNA can be extracted from samples, one can estimate biological variation by pooling multiple independent samples [20].

A variety of factors can contribute to differences in gene expression among individuals. Pritchard *et al*. [21] proposed that differences in immune status may explain the 3.3% difference in gene expression among genetically identical mice. Sex explained a large portion of among-individual variation in gene expression in *Drosophila*, whereas genotype was less of an influence, and the influence of age was weak [4]. Furthermore, this type of variation can be biologically relevant. For example recent work in *Fundulus *indicates that most inter-individual variation in metabolism can be accounted for by differences in metabolic gene expression [19].

### Variation among tissues

Another important source of biological variation in gene expression is differences in expression among different tissues; 76% of genes were differentially expressed between brain, heart and liver, and expression in the liver was the most distinct compared to heart and brain. In this study, genes printed on our array are primarily enzymes functional in central metabolic pathways such as fatty-acid metabolism, glycolysis and oxidative phosphorylation. Of the oxidative phosphorylation genes differentially expressed between tissues, 92% were more highly expressed in heart or brain than in liver (Figure 3). The primary purpose of the heart is to act as a pump, and contraction is highly dependent on oxidative metabolism [22]. The metabolic rate in the brain is 7.5 times the average rate in the rest of the body [23]. High metabolic demand in the brain supports pumping of ions across neuronal membranes during action potentials and metabolism is primarily oxidative. Mitochondria are the principal sites for oxidative phosphorylation, and are most numerous in heart, brain and skeletal muscle cells. The liver, in contrast, is much more functionally diverse, as it is involved in carbohydrate storage, synthesis of proteins, glucose, fatty acids, cholesterol and lipids, and metabolism of xenobiotics and endogenous compounds, and has a relatively low respiration rate. Accordingly, transcripts of genes functional in oxidative phorphorylation appear to represent a much smaller portion of the cell's RNA transcripts in liver tissues than in the heart or brain. In addition, genes involved in fatty acid and phospholipid synthesis were more highly expressed in liver than the other tissues. Differences in expression among tissues detected using our array appear to reflect differences in the metabolic status of brain, heart, and liver. Because data presented here support well established patterns of metabolism, they suggest that measuring mRNA expression using microarrays accurately reflects changes in proteins and their phenotypic effect.

Many microarray studies have used expression levels of 'housekeeping' genes as an internal control for comparisons among arrays, individuals and treatments. Housekeeping genes may be defined as those that are involved in routine cellular metabolism and always expressed in all cells. Accordingly, many, if not most, of the genes studied here could be considered housekeeping genes. Nearly half of these genes were expressed at different levels between individuals, with fold differences ranging from 1.2- to 5-fold and *p*-values ranging down to 10^-7^. Lee *et al*. [24] applied ANOVA to screen four previously published datasets for housekeeping genes across a variety of biological contexts. They found that all genes that are commonly used as controls had fold changes ranging from greater than 2.0 to more than 300 within at least one dataset, and coefficients of variation were concordantly high, reflecting high variance in expression of these genes. It appears that upon application of ANOVA, statistically significant differences in expression of housekeeping genes can be detected among individuals and across different biological contexts, and scaling for differences among arrays using expression levels of these genes ought to be approached with caution.

Although genes differentially expressed among tissues reflect their different metabolic requirements, it should be noted that the purpose of the current study was not to comprehensively identify suites of genes responsible for functional differences between tissues. The relatively small number of printed probes was useful for a high degree of technical replication, and obviously represents a small portion of the expressed genes. However, this approach shows that highly significant differences in gene expression among tissues may be apparent but not consistent among closely related taxa. Therefore, highly significant differences in gene expression found only within a single population may not necessarily represent genes relevant to general functional or morphological differences between tissues.

### Variation among taxa

Although the pattern of metabolic gene expression among tissues reflects established patterns of tissue-specific metabolism, there is additional variation due to population. It should be noted that the split-plot statistical design is not as powerful for detecting among-block differences (among populations) as for detecting differences among split-plot factors [18]. We detected 3% of genes (6 of 192) differentially expressed among populations. This proportion is similar to that detected in a previous study [3] in which 2.6% of genes were differentially expressed between Maine and Georgia *Fundulus *hearts. Similarly, approximately 1% of genes were differentially expressed in brain tissue among inbred strains of mice [2]. Differences in gene expression are to be expected among taxa (phylogenetically distinct groups of organisms which may include strains, populations or species), with the majority of differences most likely to be attributable to random genetic drift. For more distantly related groups, one would expect expression patterns to be more divergent than for closely related groups. Indeed, expression patterns between humans and chimpanzees are more similar than those between humans and orangutans, and similar results were obtained from comparisons among three mouse species [5,6].

An unexpected finding is that the tissue-specific differences depend on which population was assayed. Differences in gene expression are expected between tissues because of functional divergence and between populations because of neutral genetic divergence. In addition, one might expect that the number of genes significantly different between populations would depend on the tissue. One might also expect tissue-specific differences to be consistent in all taxa. Yet our data indicate that tissue-specific expression patterns are not fixed within a species. The genes for which expression is significantly different between tissues are not all the same in all three populations. Of the 128 genes that have tissue-specific patterns of expression in any population, 37% are tissue-specific in only one of the three populations and 32% are found in only two of the three populations. Overall, it would appear that only 31% of tissue-specific differences in gene expression are consistent among all populations of *F. heteroclitus*. One needs to be careful about this interpretation, however. Our emphasis was not to specifically identify genes that have significant interaction between tissue and population. Rather, we emphasize that genes detected as tissue specific will vary from one population to another, and most microarray studies measure treatment-specific expression patterns in only one population of test organism. Because inter-individual variation is high, it is probable that inclusion of more replicate individuals in each group would increase the sensitivity of ANOVA, and the number of genes that distinguish tissues consistently in all populations may change.

The consistent tissue-specific differences still support expectations based on the metabolic requirements of each tissue (for example, genes involved in oxidative phosphorylation were more highly expressed in heart and brain, and those involved in fatty-acid and lipid metabolism were more highly expressed in the liver; Figure 7a). Accordingly, those differences in expression that are consistent across several groups of organisms are most likely to account for functional and morphological differences among tissues, emphasizing that this type of comparative approach may be powerful for testing the biological relevance of other functional traits. For example, expression differences between diseased and non-diseased tissues may vary among mouse strains, so that the subset of differences that are consistent across strains are more likely to be functionally related to the diseased state.

Our data suggest that many of the differences in gene expression detected between experimental groups may be of little functional importance because they vary among taxa. We suggest that patterns of expression that are consistent in different populations are more likely to be functionally important. Elucidation of adaptively important variation, such as variation related to antibiotics, pesticides or temperature adaptation, may also benefit from such a comparative approach that screens for conserved patterns. However, there is the possibility that partitioning of genetic polymorphisms among populations may allow distinct groups of organisms to reach different physiological or biochemical solutions to the same biological challenges. For example, patterns of polymorphism in a gene that regulates coat color in mammals indicated recent directional selection and was associated with coat color in one pocket mouse population, but not in a second population [25]. Other loci were probably responsible for adaptive variation in coat color in the second population.

## Conclusions

These data indicate high variation in metabolic gene expression among individuals and thus expression of these housekeeping genes is unreliable as an internal control or as a method of normalization across samples. Second, concordance between tissue-specific expression patterns and established metabolic functions of brain, heart and liver indicate that measuring mRNA levels accurately reflects physiological status. Furthermore, since many metabolic genes differ in expression among brain, heart and liver, those studies using whole organisms need to rule out whether changes in expression reflect differences in the proportions of various tissues among samples. Finally, studies seeking to identify patterns of gene expression related to physiological states, such as disease or toxic stress, must consider both variation between individuals and differences between populations. Because of this biological variation, not all differences between treatments in any one population of test organism are likely to be generally relevant. We suggest that conserved patterns of treatment-specific gene expression among taxa are most likely to be functionally related to the physiological state in question.

## Methods and materials

### Animals and maintenance

Teleost fish *Fundulus heteroclitus *were collected from the field by seine and minnow trap in June 2003, transported to the University of Miami RSMAS laboratory under controlled temperature and aeration conditions, and acclimated to common conditions (20°C, 15 parts per thousand salinity) in recirculating 100-gallon tanks for at least two months before experiments. Fish were sacrificed by cervical dislocation and tissues were excised and stored in RNAlater (Ambion) at -20°C. Fish were collected at Wiscasset, Maine; Stone Harbor, New Jersey, and Sapelo Island, Georgia. Only healthy male fish were used for the following experiments.

### Microarrays

Microarrays were printed using 192 cDNAs from a *F. heteroclitus *cardiac library encoding essential proteins for cellular metabolism [26]. These cDNAs were a subset of over 40,000 expressed sequences in our online database Funnybase [27]. These 192 cDNAs were amplified with amine-linked primers and printed on 3-D Link Activated slides (Surmodics) using a SpotArray Enterprise piezoelectric microarray printer (PerkinElmer Life Sciences) at Louisiana State University. Slides were blocked following slide manufacturer protocols. The suite of 192 amplified cDNAs was printed as a group in six spatially separated replicates. Four hybridization zones of these six replicate arrays were printed per slide, with each zone set separated by a hydrophobic barrier.

### Hybridization experimental design

**Figure 8:**
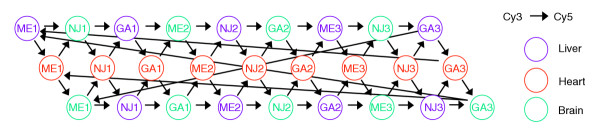
Experimental design for hybridizations. Each arrow represents an array hybridization, with the samples at arrow base and head labeled with Cy3 and Cy5, respectively. Liver, heart and brain samples are indicated as purple, red and blue circles, respectively. Three individuals were assayed per tissue and from each of three populations. ME, Maine; NJ, New Jersey; GA, Georgia.

Microarray analyses were applied to three tissues (brain, heart and liver) from three individuals collected from three populations of *F. heteroclitus*. Each of these 27 samples was measured four times, twice with Cy3 and twice with Cy5 (Figure 8). In addition, since a hybridization zone covered six replicate printed arrays, total experimental replication per sample per gene was 24-fold. A total of 108 hybridizations were performed (27 × 4), and Cy3-Cy5 hybridizations were balanced (although incompletely) among tissues and populations in a sheet-loop design (Figure 8).

### Sample preparation

RNA was extracted from tissue homogenate in a chaotropic buffer using phenol/cholorform/isoamyl alcohol. All reagents were from Sigma unless otherwise noted. Tissues were removed from RNAlater, blotted dry, and homogenized using an electric homogenizer in 400 μl chaotropic buffer (4.5 M guanidinium thiocyanate, 2% N-lauroylsarcosine, 50 mM EDTA pH 8.0, 25 mM Tris-HCl pH 7.5, 0.1 M β-mercaptoethanol, 2% antifoam A). An equal volume of 2 M sodium acetate (pH 4.0) was added to the homogenate, followed by 400 μl acidic phenol (pH 4.4), and 120 μl chloroform/isoamyl alcohol (23:1). The mixture was kept at 4°C for 10 min then centrifuged at 4°C at 16,000*g *for 20 min. Supernatant was removed and combined with 400 μl isopropanol, stored at -20°C for 30 min, then centrifuged at 4°C at 16,000*g *for 30 min. The remaining RNA pellet was rinsed twice with 400 μl of 70% ethanol, then further purified using the Qiagen RNeasy Mini kit (Qiagen) following the manufacturer's protocols. Purified RNA was quantified spectrophotometrically, and RNA quality was assessed using the Agilent 2100 Bioanalyzer. RNA was stored in 1/10 volumes 3 M sodium acetate and 2.5 volumes 100% ethanol at -20°C.

RNA for hybridization was prepared by amplification using a modified Eberwine protocol [28]. The Ambion Amino Allyl MessageAmp aRNA Kit was used (according to manufacturer's protocols) to copy template RNA by T7 amplification following incorporation of a T7 promoter, resulting in amplified template in the form of antisense RNA. Amino-allyl UTP was incorporated into targets during T7 transcription, and resulting amino-allyl antisenseRNA was coupled to Cy3 and Cy5 dyes (Amersham Biosciences).

### Hybridization

Labeled aRNA aliquots of the two individual samples for each hybridization (18 pmol each of Cy3 and Cy5) were vacuum dried together and resuspended in 12 μl hybridization buffer (final concentration of each labeled sample = 1.5 pmol/μl). Hybridization buffer consisted of 5 × SSPE, 1% SDS, 50% formamide, 1 mg/ml poly(A), 1 mg/ml sheared herring sperm carrier DNA, and 1 mg/ml BSA. Slides were washed in sodium borohydride solution according to Raghavachari *et al*. [29] to reduce autofluorescence. Following rinsing, slides were boiled for 2 min and spin-dried in a centrifuge at 800 rpm for 3 min. Samples (12 μl) were heated to 90°C for 2 min, quick cooled to 42°C, applied to slide (hybridization zone area was 350 mm^2^), and covered with a coverslip. Slides were placed in an airtight chamber humidified with paper soaked in 1 × SSC buffer and incubated 12-18 h at 42°C. Following hybridization, slides were scanned using the Packard Bioscience ScanArray Express microarray scanner (PerkinElmer Life Sciences). Resulting .tiff images were imported into spot grids built in ImaGene (Biodiscovery) for each array, and spot signals were collected as fluorescence intensities for each dye channel.

### Data processing and statistical analysis

**Figure 9:**
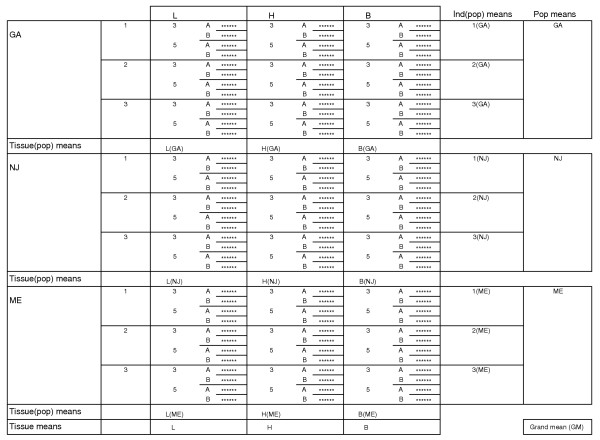
Split-plot ANOVA statistical design. Populations (ME, Maine; NJ, New Jersey; GA, Georgia) are treated as blocks, replicate individuals within each population (1, 2 and 3) as plots, and tissue (L, liver; H, heart; B, brain) within an individual as the split-plot factor. Nested within each tissue-by-individual sample are technical replicates including two dyes (Cy3 and Cy5) within each sample, two replicate hybridizations (A and B) per dye, and six replicate spots per hybridization. GM, grand mean.

Raw data were first sum normalized [30], which involves summing the total signal from each replicate array to the same value. Then spatial bias on each array was smoothed using a lowess transformation in MAANOVA Version 0.93-2 for R [31]. Other methods of normalization have also been proposed [32-34]. Log_2 _values of lowess-transformed sum-normalized data were used for all subsequent statistical analyses. MIAME-compliant data [35] have been submitted to the Gene Expression Omnibus as accession number GLP1224. Data were analyzed in a split-plot ANOVA design with population as blocks and tissues as split-plot factors using scripts written in MatLab Version 6 (The MathWorks). MatLab code is available upon request from the authors. Nested within tissue-by-population samples were technical replicates. Replicate spots within hybridization (six), replicate hybridizations per labeling (two) and replicate labelings per sample (two; Cy3 and Cy5) represent the three levels of technical variance nested within the tissue-by-population sample. The ANOVA structure is presented in Figure 9 and Table 2, and the model can be written as:

*y *= grand mean + population + tissue + population-tissue interaction + individual in population + tissue-by-individual within population + dye within individual + hybridization within dye + spot within hybridization

where *y *is the normalized log_2 _expression and individual in population and tissue-by-individual within population are random effects. To test for differences among multiple means (for example, among population and tissue groups), and to correct for multiple comparisons, the T-method [36] was applied. The T-method calculates the minimum significant range defined as

MSR = Q_α[*kv*] _× SE

where the critical value Q_α[*kv*] _is the studentized range [37], *k *= number of groups in the comparison (for example, if comparisons are among tissues then *k *= 3), v = degrees of freedom of MS_tissue-by-individual within population_, and SE is the standard error among tissue-by-individual samples within populations. The T-method following ANOVA was used to identify genes differentially expressed among tissues in each population. These data were then used to contrast tissue-specific and population-specific expression patterns. Robustness of ANOVA data was tested using a permutation test; means for the 27 biological samples were randomly permuted 1,000 times between population and tissue and test statistics were recalculated for differences among populations, tissues and tissue-by-population interaction. Agreement between ANOVA and permutation test results would indicate the robustness of the ANOVA model. Finally, in order to graphically illustrate expression similarity among tissues, expression distance between samples was calculated as the sum of differences of log_2 _expression values over all genes, and neighbor-joining trees of global similarity of expression patterns among tissues (L, liver; H, heart; B, brain) were constructed [38] for each population.

## Additional data files

The following additional data are available with the online version of this paper. Additional data file 1 lists the results from statistical analyses for all genes. Listed for each gene are *p*-values associated with statistical tests for differences in expression between populations, tissues, tissue-by-population interaction, and among individuals within populations. Also listed are mean expression for each sample, and columns comparing differences in expression between tissues within each population. Final columns tabulate whether a tissue difference was detected for each comparison, whether this difference was consistent between populations, and whether significant interaction was detected for that gene.

## Supplementary Material

Additional data file 1 The results from statistical analyses for all genes Click here for additional data file

## Figures and Tables

**Table 1:** Identity of tissue-specific genes with expression patterns consistent in all three populations, and those inconsistent in all three populations

Gene (see Figure 7)	Grid	Short name	Swiss-Prot name
**Consistent - oxidative phosphorylation**			
a	E8	Aldo keto reductase 1 A1	Aldo-keto reductase family 1 member A1 (aldehyde reductase)
b	E7	Aldo keto reductase 1 D1	Aldo-keto reductase family 1 member D1; steroid-5-beta-reductase beta polypeptide 1 (3-oxo-5 beta-steroid delta 4-dehydrogenase beta 1); steroid 5-beta-reductase
c	F1	G3PDH	Glyceraldehyde-3-phosphate dehydrogenase (GAPDH)
d	D10	Glucose 6 phosphatase	Glucose-6-phosphatase (G6PASE)
e	H3	Pyruvate kinase muscle	Pyruvate kinase (muscle isozyme)
f	G10	Pyruvate kinase R	Pyruvate kinase isoform R (erythroid)
g	I6	NADH dehydrb 6 (17 kD)	NADH dehydrogenase (ubiquinone) 1 beta subcomplex 6 (17 kD B17)
h	G4	NADH Ubiq Oxi ASHI	NADH-ubiquinone oxidoreductase ASHI subunit precursor (complex I-ASHI) (CI-ASHI)
i	M4	NADH Ubiq Oxi MNLL	NADH-ubiquinone oxidoreductase MNLL subunit (complex I-MNLL) (CI-MNLL)
j	L1	ATP syn H^+ ^FO c 9 2	ATP synthase H^+ ^transporting mitochondrial F0 complex subunit c (subunit 9) isoform 2
k	P3	ATP syn H^+ ^FO F6	ATP synthase H^+ ^transporting mitochondrial F0 complex subunit F6; coupling factor 6
l	I9	Cyto C oxi III	Cytochrome c oxidase subunit III
m	J10	Cyto C oxi VA	Cytochrome C oxidase polypeptide VA
n	N8	Cyto C oxi VIa	Cytochrome c oxidase subunit VIa precursor polypeptide 2
o	J5	Cyto C oxi VIIC	Cytochrome C oxidase polypeptide VIIC precursor (VIIIA)
p	K12	Cyto C oxi VIIIb	Cytochrome c oxidase subunit VIIIb
			
**Consistent - other metabolism**			
q	H12	Isocitrate dehyd 2	Isocitrate dehydrogenase 2 (mitochondrial IDH2)
r	A9	PEP carboxykinase	PEP carboxykinase phosphoenolpyruvate carboxykinase
s	D8	Fatty acid binding liver basic	Liver-basic fatty acid binding protein (LB-FABP)
t	B12	Delta 6 fatty acid desaturase	Delta-6 fatty acid desaturase
u	H1	Triglyceride lipase triacylglycerol	Triglyceride lipase triacylglycerol
v	I5	Glycerol kinase	Glycerol kinase
w	M10	Lipoprotein lipase	Lipoprotein lipase
x	P9	Phospholipase XIII A2	Group XIII secreted phospholipase A2
y	F4	Cystathionine beta synthase	Cystathionine-beta-synthase
z	K11	Cold inducible RNA binding	Cold inducible RNA-binding protein; (CIRBP) glycine-rich RNA binding protein;
aa	F2	Hepatocyte nuclear F 4 A	Hepatocyte nuclear factor 4-alpha (HNF-4-alpha) (transcription factor HNF-4)
bb	M1	p450 2P1 (CYP2P1)	Cytochrome P450 2P1 (CYP2P1)
cc	D6	Glutathione peroxidase 4	Glutathione peroxidase 4 (phospholipid hydroperoxidase)
dd	O11	Methylmalonate semialdehyde dehyd	Methylmalonate-semialdehyde dehydrogenase (acylating)
ee	N7	Phosphatidylcholine sterol acyltrans	Phosphatidylcholine-sterol acyltransferase
ff	B1	Prostaglandin D syn	Prostaglandin D synthase
			
**Inconsistent - oxidative phosphorylation**			
gg	A6	ADH class II mito	Aldehyde dehydrogenase, mitochondrial precursor (ALDH class 2)
hh	E12	Aldolase 1 A	Aldolase 1 A. muscle
ii	A2	Enolase beta muscle	enolase (beta muscle specific)
jj	F7	LDHB	lactate dehydrogenase B (LDHB)
kk	O6	PFK	6-phosphofructokinase
ll	K4	NADH dehyd MLRQ	NADH dehydrogenase (ubiquinone) MLRQ subunit (complex I-MLRQ)
mm	L9	NADH dehyd I	NADH dehydrogenase subunit 1
nn	C6	NADH dehydr a 1 (7.5 kD MWFE)	NADH dehydrogenase (ubiquinone) 1 alpha subcomplex 1 (7.5 kD MWFE)
oo	E6	NADH dehydr a 9 (39 kD)	NADH dehydrogenase (ubiquinone) 1 alpha subcomplex 9 (39 kD)
pp	M6	ATP syn B	ATP synthase subunit B
			
**Inconsistent - other metabolism**			
qq	C7	Transketolase	Transketolase
rr	H8	Fatty acid binding 7 brain	Fatty acid binding protein 7 brain (B-FABP)
ss	A3	Fatty acid binding H6	Fatty acid binding protein H6-isoform
tt	O10	Fatty acid binding heart	Heart-type fatty acid-binding protein (H-FABP)
uu	D9	Fatty acid syn	Fatty acid synthase
vv	F9	Glutamate decarboxylase	Glutamate decarboxylase

Letters in the first column refer to genes illustrated in Figure 7; the grid column identifies genes as reported in our data entry to the NCBI Gene Expression Omnibus (GLP1224). Gene identities are listed as those identified by Swiss-Prot and as shortened names, and grouped as genes involved in oxidative phosphorylation or in other biochemical pathways.

**Table 2:** Sources of variance and calculation of variables for the split-plot ANOVA statistical design [18]

Source of variance	df		Sum of squares		Fs
Among populations	P - 1	2	SS_P	I × T × Σ(popmeans - GM)^2^	MS_P/MS_I(P)*
Among tissues	T - 1	2	SS_T	I × P × Σ(tissuemeans - GM)^2^	MS_T/MS_TI(P)^†^
Interaction	(P - 1) × (T - 1)	4	SS_PT	I × Σ(tissue(population)means - popmeans - tissuemeans + GM)^2^	MS_PT/MS_TI(P)^‡^
Among inviduals in population	P(I - 1)	6	SS_I(P)	T × Σ(ind(population)means - popmeans)^2^	
Tissue-by-individual in population	P × (T - 1) × (I-1)	12	SS_TI(P)	Σ(samplemean - tissue(population)mean - ind(population)mean + popmean)^2^	MS_TI(P)/MS_dye^§^
Dye within individuals	P × T × I × (D - 1)	27	SS D(I)		
Replicate hybridization in dye	P × T × I × D × (H - 1)	54			
Spot in replicate hybridization	P × T × I × D × H × (S - 1)	540			
Total	P × T × I × D × H × S - 1	647			
